# Atlanta Medical College

**Published:** 1871-05

**Authors:** 


### Editorial and Miscellaneous.

*Atlanta Medical College.*

The exercises are progressing in a satisfactory manner, notwithstanding the gloom which overspread the institution at the opening of the present course. The death of Prof. Jones, occurring at the very eve of the session, not only filled with sadness all connected with the College, but left vacant an important chair. The Prudential Committee of the Board of Trustees, however, came forward promptly to the relief of this embarrassment, and authorized the appointment, for the course, of Dr. E. D. Withers. In him the class has already the evidence of eminent scientific attainments, and a happy faculty for thorough and correct teaching. While the Faculty and class mourn the loss of a cherished colleague and teacher, they feel grateful for the promptness with which the Trustees have supplied his place with one every way competent for the position.

The Chair of Physiology, which had also been left vacant, was at the same time supplied by the appointment of Dr. William A. Love, whose thorough knowledge of the science of life, and his talent for instruction, will insure a successful and satisfactory course in this department.

The Clinique, in connection with the College Dispensary, is conducted by members of the Faculty, and affords an amount of material quite sufficient to occupy an hour, which is devoted each day to the practical studies of diagnosis and the therapeutical effects of remedies.

In addition to this, two or three surgical clinics a week will be afforded throughout the session, and an hour every Monday afternoon devoted to the practical study of female disease, exclusively. In the daily morning clinic, diseases peculiar to females are also presented, on Monday and Friday; so that the various forms of chronic uterine disease can be very thoroughly studied during the course of lectures.

Efforts are being made by the Faculty to make the course of instruction more extensive and thorough than usual, and the facilities for acquiring a medical education equal to any institution in this country.

Microscopy and bandaging are taught practically during such leisure hours as can be conveniently devoted to these studies.

